# This I Believe: Gaining New Insights Through Integrating “Old” Data

**DOI:** 10.3389/fgene.2012.00137

**Published:** 2012-07-31

**Authors:** Ruth J. F. Loos, Eric E. Schadt

**Affiliations:** ^1^Charles R. Bronfman Institute of Personalized Medicine, Institute of Child Health and Development, Department of Preventive Medicine, Mount Sinai School of MedicineNew York, NY, USA; ^2^Department of Genetics and Genomic Sciences, Institute for Genomics and Multiscale Biology, Mount Sinai School of MedicineNew York, NY, USA

Genome-wide association studies (GWAS) have been tremendously effective in identifying genetic loci unequivocally associated with a myriad of common traits and diseases (Hindorff et al., 2009, 2012). The latest update of the NHGRI GWAS catalog reported more than 1,600 SNPs associated with any of the nearly 250 traits at genome-wide significance (Hindorff et al., 2012). Proponents praise the genome-wide association approach for having accelerated gene discovery immensely since their debut in 2005, before which human geneticists relied mainly on the less fruitful candidate gene and genome-wide linkage studies. Skeptics question the value of GWAS discoveries, as relatively few have increased our insights in to the biology underlying disease and even fewer have been successfully translated into mainstream health care. Both arguments hold water. Further gene discovery should be applauded and will require ever-growing sample sizes, ethnically diverse populations, more accurately defined phenotypes, accounting for environmental influences, and/or more refined genomic information. The falling cost of whole-exome and genome sequencing and the advent of new technology, will likely further boost gene discovery in the coming years. But now is also a good time to address the skeptics’ plea for proof that GWAS discoveries, and genetics in general, have the potential to provide valuable etiological insights and to eventually improve disease prediction, prevention, and treatment.

We believe that this, at least in part, can be achieved by taking time to look back at the discoveries that have been made over the past few years and by integrating this information in to a more comprehensive and dynamic manner. It is encouraging to see that there is indeed a growing interest in putting the pieces of the puzzle together in several fields, both the phenotypic and genomic components.

Progress has been made in the field of autoimmune disease, for which it became clear early on in the GWAS era that several autoimmune conditions had a common genetic background. So far, at least 140 genetic loci have been identified to be associated with immune-mediated and autoimmune disease, many of which (>40%) are shared across multiple diseases, yet the magnitude of their effect often differs by disease (Cotsapas et al., 2011). By considering all established loci of seven immune-mediated and autoimmune diseases in a cross-phenotype meta-analyses, several clusters of genotype-phenotype associations were identified that appeared to underlie the same conditions, whereas other previously presumed shared loci turned out to have distinct effects on separate conditions (Cotsapas et al., 2011). The latter observation highlights the challenges to disentangle whether apparently shared genes and variants affect the multiple traits through a truly shared biology or rather through independent effects. The genetic loci for most other disease groups have not yet been considered in a such systematic integrated way. For example, more than 150 genetic loci have been identified for various cancers, and while at least seven regions were found to be associated with multiple cancers (Chung and Chanock 2011), potentially more such genetic nexus regions could be identified if all association results were evaluated simultaneously. Obesity and metabolically related traits is another field where the systematic study of shared genetic variation could elucidate new biological mechanisms. Of the 95 loci identified for lipids, some associate with all traits – i.e., triglyceride, HDL-cholesterol, LDL-cholesterol, and total cholesterol levels, whereas other loci affect only one trait at a time (Teslovich et al., 2010). Finding patterns of association across traits may reveal distinct physiological pathways, which could contribute to more targeted treatment. Other interesting observations have been made for type 2 diabetes. While the definition of type 2 diabetes is based on glucose levels, some genetic loci that are associated with fasting or 2H-glucose levels are not associated with type 2 diabetes and vice versa (Dupuis et al., 2010; Saxena et al., 2010). The reasons for this apparent contradiction have not yet received extensive attention. Intriguing was also the association pattern of a variant near *IRS1*, of which the major allele was associated with lower body fat percentage, but also with increased risk of type 2 diabetes and cardiovascular disease, as well as with a poor lipid profile (Kilpelainen et al., 2011). Further analyses showed that the variant decreased subcutaneous fat, but not the more harmful visceral fat. Many observations of a common genetic background to a cluster of diseases are still andecdotical and much is to be gained from more systematic approaches. New methods are being developed (Cotsapas et al., 2011; Pendergrass et al., 2011) and consortia-of-consortia are formed (e.g., the XC-Pleiotropy Group) to search for such shared genetic associations across multiple traits simultaneously in a systematic manner.

Besides integrating phenotypic information, progress has also been made on integrating genomic components. Despite the complexity of common human diseases involving large numbers of genetic and environmental effects driving changes at the molecular, cellular, organ, organism, and even community levels, research in the context of large-scale high dimensional *omics* data have tended to focus on single data dimensions, whether carrying out GWAS, constructing co-expression networks based on gene expression data, or constructing protein interaction networks based on protein–protein interaction data. While we can achieve some understanding of disease in this way, progress is necessarily limited because none of the dimensions on their own provide a complete enough context within which to interpret results fully. This type of limitation has become apparent in GWAS or whole-exome or genome sequencing studies, where thousands of highly replicated loci have been identified and highly replicated as associated with disease, but our understanding of disease is still limited because the genetic loci do not necessarily inform on the gene affected, on how gene function is altered, or more generally, how the biological processes involving a given gene are altered at particular points of time or in particular contexts (Altshuler et al., 2008; Chen et al., 2008; Emilsson et al., 2008; Witte, 2010). It is apparent that if different biological data dimensions could be formally considered simultaneously, we would achieve a more complete understanding of biological systems (Chen et al., 2008; Emilsson et al., 2008; Schadt et al., 2008; Hsu et al., 2010; Zhong et al., 2010a).

The integration of more dynamic, functional data like gene expression, protein expression, protein state, metabolite levels, protein-DNA binding, and so on can directly elucidate the molecular processes underlying the pathophysiological states that define disease. The identification of molecular features like RNA, proteins, and metabolites that mediate the flow of information from DNA to disease can directly implicate specific genes and the biological processes in which they operate in disease. Studies that seek to integrate multiple dimensions of data at the population level have the potential to reveal the network of disease causing genes acting together in coherent networks to drive disease (Chen et al., 2008; Emilsson et al., 2008; Schadt et al., 2008; Zhong et al., 2010a; Schadt and Bjorkegren 2012; Zhu et al., 2012), where such empirical networks expand upon our understanding of more canonical pathways that today provide a sparse framework in which to understand disease related processes (Tu et al., 2009; Yang et al., 2009, 2010a,b; Zhong et al., 2010a,b). The types of integrative algorithms that provide for mathematical frameworks that can simultaneously consider many different dimensions of data are beginning to emerge (Zhu et al., 2012), promising to provide a better functional understanding of disease causing genes and the contexts in which they operate, a necessary prerequisite for developing better ways of treating and diagnosing disease.

Putting all available pieces of the puzzle together, will provide us with the first impressions of the emerging larger pictures that will be needed to guide the completion of the full movie.
